# Interactions of Laurylated and Myristoylated KR12 Fragment of the LL37 Peptide with Polyoxidovanadates

**DOI:** 10.3390/molecules30071589

**Published:** 2025-04-02

**Authors:** Martyna Kapica, Elżbieta Kamysz, Ola Grabowska, Aleksandra Tesmar, Marek Pająk, Katarzyna Chmur, Jakub Brzeski, Sergey A. Samsonov, Dariusz Wyrzykowski

**Affiliations:** 1 Faculty of Chemistry, University of Gdańsk, Wita Stwosza 63, 80-308 Gdańsk, Poland; martyna.kapica@phdstud.ug.edu.pl (M.K.); elzbieta.kamysz@ug.edu.pl (E.K.); ola.grabowska@ug.edu.pl (O.G.); aleksandra.tesmar@ug.edu.pl (A.T.); katarzyna.chmur@ug.edu.pl (K.C.); jakub.brzeski@ug.edu.pl (J.B.); 2 Department of Physical and Biocoordination Chemistry, Medical University of Lodz, Muszyńskiego 1, 90-151 Lodz, Poland; marek.pajak@umed.lodz.pl

**Keywords:** polyoxidometalates, vanadium, lipopeptide, peptide–ligand interactions, isothermal titration calorimetry, molecular dynamics

## Abstract

Isothermal titration calorimetry (ITC), circular dichroism (CD) spectroscopy, and molecular dynamics simulations were applied to describe interactions between lipopeptides and decavanadate ions ([V_10_O_28_]^6−^). The selected lipopeptides are conjugates of the amide of the KR12 peptide, the smallest antimicrobial peptide derived from human cathelicidin LL-37, with lauric acid (C12-KR12) and myristic acid (C14-KR12). The smaller sizes of C12-KR12 and C14-KR12 compared to proteins allow for the rigorous characterization of their non-covalent interactions with highly negatively charged [V_10_O_28_]^6−^ ions. The stoichiometry of the resulting decavanadate–peptide complexes and the thermodynamic parameters (Δ*G*, Δ*H*, and TΔ*S*) of the interactions were determined. The ITC results, supported by the MD simulation, showed that the binding of cationic lipopeptides for decavanadate is rather non-specific and is driven by enthalpic contributions resulting from electrostatic interactions between the positively charged residues of the peptides and the anionic decavanadate. Furthermore, the influence of temperature and the interactions with decavanadate ions on the stability of the α-helical structure of the lipopeptides were assessed based on CD spectra. Under the experimental conditions (50 mM sodium cacodylate buffer, pH 5), the peptides adopt an α-helical conformation, with C14-KR12 showing greater thermal stability. The interactions with vanadium species disrupt the α-helical structure and reduce its thermal stability.

## 1. Introduction

Polyoxidometalates (POMs) are anionic polynuclear metal–oxide clusters composed predominantly of tungsten(V), molybdenum(V), vanadium(V), or niobium(V). However, the structure of POMs can also incorporate other transition metal ions, such as Co(II) or Mn(II), and various heteroions, including phosphorus(V) and arsenic(V) [1]. The unique structural features and physicochemical properties of polyoxidometalates have recently attracted significant interest for their potential applications in various fields. They are being explored for their therapeutic potential in medicine as candidates for anti-diabetic, antibacterial, anti-protozoal, antiviral, and anticancer agents [2,3]. In addition, POMs are gaining attention mainly as catalysts in the fields of engineering, nanotechnology [4,5,6], and materials science [7,8,9]. A separate subgroup of polyoxidometalates includes vanadium(V)-containing structural units, namely polyoxidovanadates (POVs). In aqueous systems, different types of vanadate(V) species, namely monomeric ([VO_4_]^3−^), dimeric ([V_2_O_7_]^4−^), tetrameric ([V_4_O_12_]^4−^), and decameric ([V_10_O_28_]^6−^), occur simultaneously in equilibrium. The average concentration of vanadium in the human body is about 14 ug per 1 kg of body weight, with an average concentration in blood plasma of about 45 nM. Interestingly, some marine organisms, particularly tunicates, can accumulate vanadium in blood cells, reaching extreme vanadium concentrations of 350 mM [10]. An excellent review by J.C. Pessoa et al. [11] highlights the significant influence of experimental conditions, such as the pH of the environment and the total concentration of vanadium(V) ions, on the equilibrium between different species. Under physiological conditions, low concentrations of vanadium(V) do not promote the oligomerization of vanadium(V) species. However, under certain conditions, particularly when the pH is below 5.5, decavanadates ([H_n_V_10_O_28_]^6−n^) become important species [11].

In biological systems, decavanadates are found in a range of tissues and cells, where they interact with various bioligands such as enzymes, polyphosphates, nucleotides, and DNA [12]. These interactions can affect cellular signaling pathways. They can also be found in mitochondria and the endoplasmic reticulum, where they disrupt cellular metabolism and affect energy balance [12]. The investigation of the interactions of vanadate(V) species with different bioligands is challenging, as specific experimental conditions can strongly influence these interactions. For these reasons, it is essential to consider these factors when designing experiments involving POVs. Recent studies on the interactions between POVs and proteins have shown that the affinity of POVs for proteins is determined by hydrogen bonds, electrostatic and van der Waals forces [1]. Crystallographic and computational studies indicate that POVs, due to their high negative charge, prefer to bind to positively charged protein surfaces or in the protein core. The positively charged side chains of the lysine, arginine, and histidine amino acid residues around the [H_n_V_10_O_28_]^6−n^ anion stabilize the resulting POV–protein complexes [1].

In contrast to proteins, relatively little attention so far has been paid to the interaction of POVs with peptides [13,14]. The proven ability of POVs to interact with proteins suggests that the presence of decavanadates in biological systems also affects interactions with biologically relevant peptides [12,14,15]. POV–peptide interactions can affect the structure by disrupting or stabilizing specific secondary structures of the peptide, leading to the alteration or modulation of its functions. Consequently, the physicochemical characterization of POV–peptide interactions is essential for enhancing our understanding of the activity of polyoxidometalates in biological systems. Thus, this was the reason that prompted us to undertake these studies.

**Figure 1:**
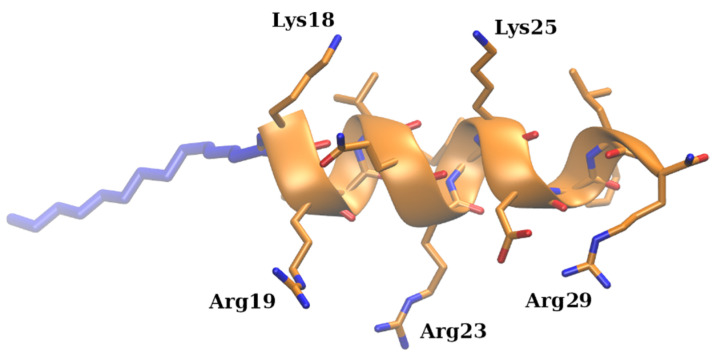
The structure of the C14-KR12 lipopeptide (the myristate chain conjugate part is shown as thick blue sticks, positively charged residues of KR12 are shown as sticks, and the secondary structure of the KR12 peptide is rendered as a cartoon).

Two lipopeptides, designated as C12-KR12 and C14-KR12 and representing the conjugation of the KR12 peptide (K^18^R^19^IVQR^23^IK^25^DFLR^29^-NH2), the smallest antimicrobial peptide derived from human cathelicidin LL-37, with lauric (C12-) and myristic (C14-) fatty acids, were selected for this study. The representative structure of C14-KR12 is shown in Figure 1.

Antimicrobial peptides (AMPs), such as LL-37, are positively charged peptides rich in arginine (R) and lysine (K) that play a crucial role in the innate immune system [16]. As a result of their specific interaction with the negatively charged bacterial membranes, they prevent the pathogens from developing resistance, which leads to the death of the pathogen. KR-12, consisting of 12 amino acid residues, is derived from the LL-37 sequence (residues 18–29) and acts on anionic bacterial membranes in a similar mode to LL-37. It has been reported that the antimicrobial activity of AMPs is significantly influenced by basic amino acids, which facilitate bacterial recognition through long-range electrostatic interactions and are crucial for targeting Gram-negative bacteria. In contrast, hydrophobic amino acids help anchor the peptide to bacterial membranes through van der Waals forces [16]. Recently, we have proven that modifying KR-12 by conjugating different n-alkyl acids with the N-terminus of the peptide enhances antimicrobial activity against both Gram-negative and Gram-positive bacteria [17]. Investigation of the interactions between cationic peptides such as AMPs and decavanadates that represent a simple model of negatively charged molecules can provide some insights into the molecular recognition and subsequent binding of these peptides to negatively charged species such as negatively charged bacterial membranes or DNA.

In this contribution, we present, for the first time, the physicochemical principles underlying the interactions between decavanadates ([H_n_V_10_O_28_]^6−n^) and two lipopeptides: the laurylated and myristoylated KR12 fragments, denoted as C12-KR12 and C14-KR12, respectively. These studies can help elucidate the potential mechanisms of biological action of the compounds under investigation and improve our understanding of the fundamental biological processes in which they are involved.

## 2. Results and Discussion

### 2.1. Experimental Conditions Affect the Structure and Charge of the Investigated Molecules

**Figure 2:**
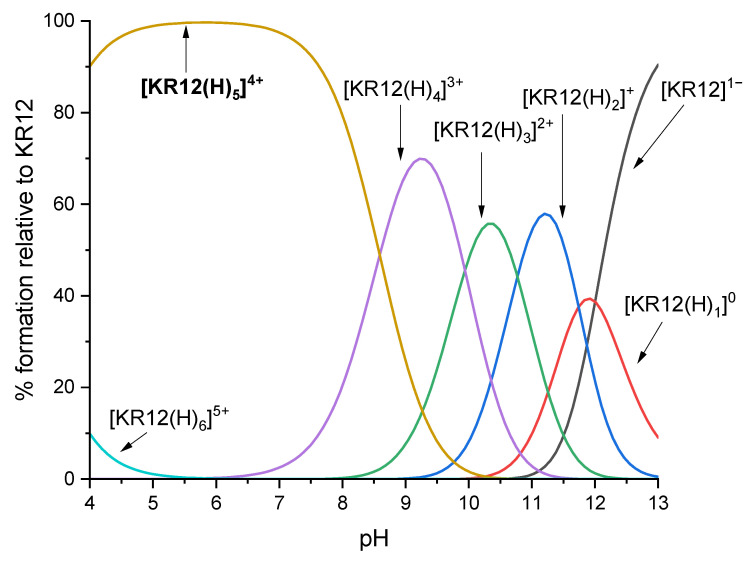
Species distribution diagram of KR12 as a function of pH (calculated based on p*K_a_* values taken from [18]).

Considering the pH-dependent equilibrium between the different species present in the aqueous solution, the interactions involving decavanadate ions were studied in a buffer solution at pH 5, where decavanadate ions predominate [1,11]. Under these conditions, the charge of the C12-KR12 and C14-KR12 peptide fragments is +4, resulting from the positively charged residues of two lysines (Lys, K) and three arginines (Arg, R), while an aspartic acid residue (Asp, D), which is deprotonated at pH 5, contributes a negative charge of −1. Figure 2 shows the species distribution diagram of the KR12 peptide constructed based on the dissociation constants previously reported in [18].

Changes in pH can cause the protonation or deprotonation of functional groups in polypeptides, altering their overall charge and affecting the stability of vanadium (V) species in the system. Consequently, the pH of a system can significantly affect the binding interactions between lipopeptides and vanadium (V) species. It is therefore crucial to control the pH in experiments with the species under investigation to gain accurate insights into their binding interactions.

### 2.2. Thermodynamic Parameters of Lipopeptide–Polyoxidovanadate Interactions

**Figure 3:**
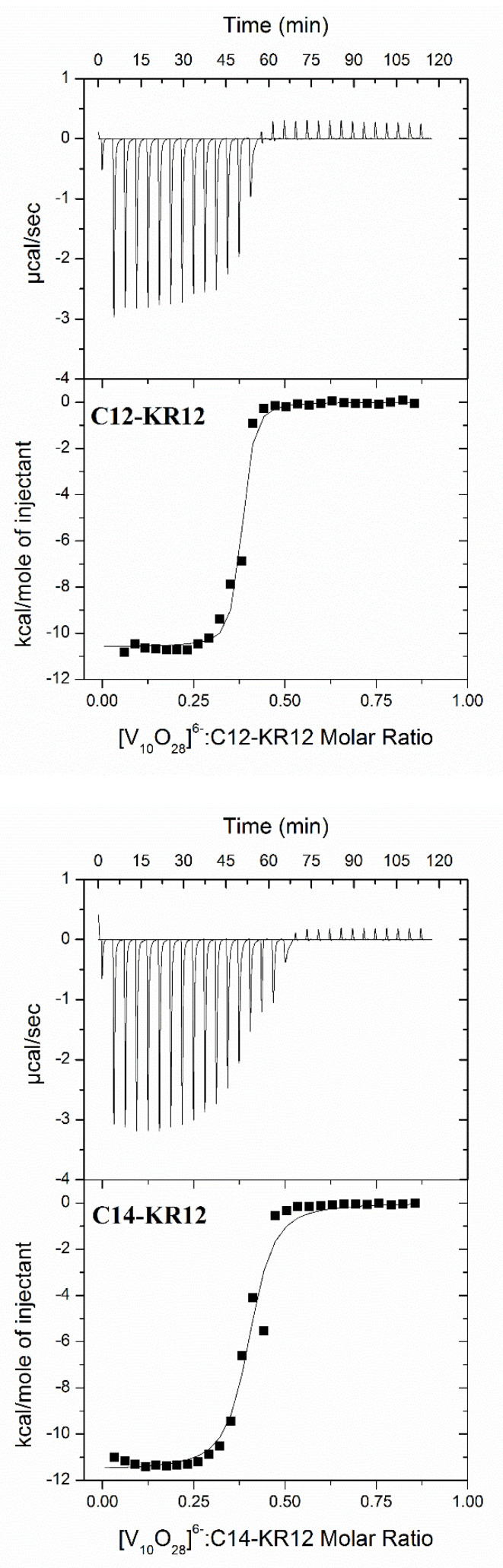
Calorimetric titration isotherms of the binding interactions of lipopeptides (C12-KR12 and C14-KR12) with decavanadates in the 50 mM sodium cacodylate buffer with a pH of 5, at 298.15 K.

The structure of POVs and the conformation of lipopeptides are highly susceptible to alteration, particularly by variations in the pH of a system and the presence of competing ligands. The affinity and the nature of the POV–lipopeptide interactions can be affected by variations in environmental conditions. As a result, the parameters obtained from isothermal titration calorimetry (ITC) depend upon the specific conditions of the experiment. Therefore, to ensure relevant analysis, comparisons of ITC results with data obtained using other techniques should only be made if the measurements were carried out under identical experimental conditions [11]. From the ITC measurements, the binding isotherms were analyzed using a model that assumes the existence of one set of binding sites, providing the best fit for the experimental data. Representative binding isotherms for the systems under study are shown in Figure 3, whereas conditional parameters of the interactions (50 mM sodium cacodylate buffer, pH 5, 298.15 K) are summarized in Table 1.

ITC studies indicate that the stoichiometry of the interaction between [V_10_O_28_]^6−^ and C12-KR12, as well as C14-KR12, is approximately 1:3. The following general equations represent the interaction under study:

1 [V_10_O_28_]^6−^ + 3 C12-KR12 = [V_10_O_28_]^6−^-{C12-KR12}**_3_** (1)

1 [V_10_O_28_]^6−^ + 3 C14-KR12 = [V_10_O_28_]^6−^-{C14-KR12}**_3_** (2)

The positive charge of the lipopeptide fragment suggests a strong affinity for anionic decavanadate species. This suggestion is confirmed by the negative values of binding enthalpy (Δ*H*_ITC_), indicating the important involvement of negatively charged [V_10_O_28_]^6−^ anions and positively charged lysine and arginine functional groups in the charge–charge electrostatic interactions and the hydrogen-bonding interactions. As a result, the formation of the investigated [V_10_O_28_]^6−^–lipopeptide complexes is primarily driven by enthalpy. The negative Δ*H*_ITC_ values compensate for the unfavorable negative contribution of the entropic factor (*T*Δ*S*_ITC_). Recently, combined experimental and theoretical studies have shown that the non-covalent interactions between decavanadates and guanidinium and spermidinium cations, which serve as small biomimetic models of the amino acids arginine and lysine, play an important role in their mutual attraction [19]. In particular, hydrogen bonds, especially N-H···O, play an important role in stabilizing the resulting adducts. Furthermore, hydrogen-bonding interactions involving the decavanadate anion and the zwitterion of the simple peptide glycylglycine (Gly-Gly) have been illustrated for solid-state salts [20]. The results obtained highlight the crucial role of lysine- and arginine-containing peptides in interactions with POVs. This finding is consistent with the results of molecular dynamics (MD) simulations, which further support the importance of these positively charged amino acid residues in mediating complex formation.

### 2.3. The Effect of Environmental Conditions on the Secondary Structure of Lipopeptides

It has previously been reported that the conformations of the investigated lipopeptides are sensitive to environmental conditions [17]. In pure aqueous solutions, C12-KR12 and C14-KR12 avoid adopting a defined conformation. In turn, the α-helix conformation of the peptides was found in the presence of micelles formed by anionic and zwitterionic membrane-mimicking surfactants, namely sodium dodecyl sulfate (SDS) and dodecylphosphocholine (DPC), as well as liposomes such as 1-palmitoyl-2-oleoyl-sn-glycero-3-phosphocholine (POPC) and 1-palmitoyl-2-oleoyl-sn-glycero-3-phosphoglycerol (POPG), which represent the models of prokaryotic and eukaryotic cell membranes [21].

In this study, we investigated the physicochemical properties of C12-KR12 and C14-KR12 under experimental conditions of a 50 mM sodium cacodylate buffer at pH 5 and 298.15 K. Circular dichroism spectroscopy revealed that both peptides exhibit α-helical conformations, with estimated α-helix contents of 36% and 49% for C12-KR12 and C14-KR12, respectively. Notably, C14-KR12 appears to be more prone to ordering than C12-KR12, suggesting a possible greater involvement of the longer hydrocarbon chain fragment (the myristoyl group, C14-) in stabilizing the α-helical conformation. This phenomenon is primarily driven by hydrophobic interactions, which stabilize the α-helical conformation, resulting in an increased α-helix content.

**Figure 4:**
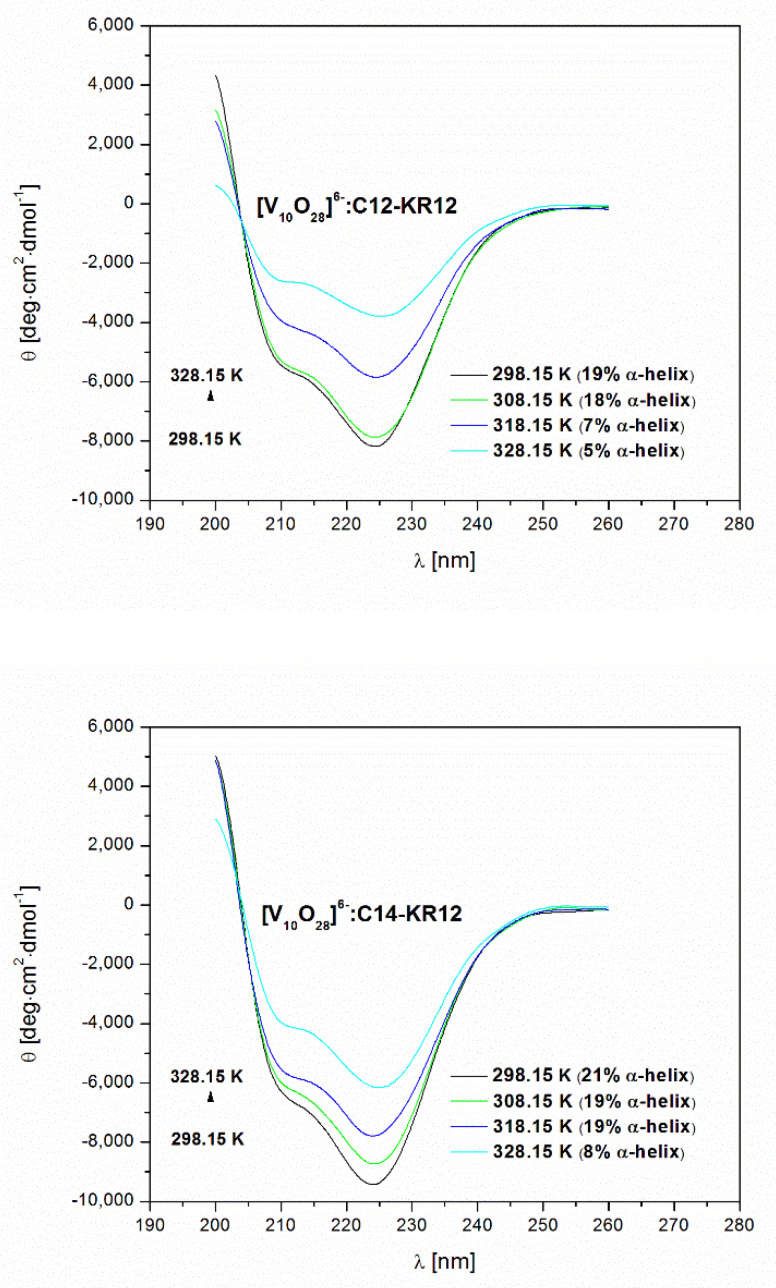
CD spectra of C12-KR12 and C14-KR12 peptides recorded in the 50 mM sodium cacodylate buffer at pH 5 and at different temperatures.

Temperature-induced conformational changes indicate that the myristoyl group exerts a greater effect on enhancing the thermal stability of the α-helical structure compared to the lauroyl group. The transitions to less ordered states are reversible processes and upon subsequent cooling, both peptides show refolding to their original α-helical conformation (Figure 4).

CD spectroscopy analysis revealed that the peptides adopt an α-helical conformation under experimental conditions and interact with POVs in this form (Figure 4 and Table 2). It is noteworthy that these interactions result in the disruption of the α-helical structure of the peptides. At 298.15 K, a significant decrease in α-helical content was observed for peptides C12-KR12 and C14-KR12 as a result of their interactions with POVs. Specifically, the α-helical content decreased from 36% to 19% for C12-KR12 and from 49% to 21% for C14-KR12 (Table 2). This finding highlights the impact of these interactions on the structural integrity of the peptides. This reduction in α-helical structure can be attributed to interactions between vanadium(V) species and positively charged side chains of lysine and arginine residues. The resulting decrease in electrostatic repulsion between these positively charged amino acids destabilizes the α-helical structure, ultimately leading to a reduction in the overall α-helical content of the peptides.

POV–peptide binding interactions not only lead to a reduction in the α-helical content of both peptides but also contribute to a reduction in the thermal stability of the secondary structure (Table 2). This destabilization suggests that the peptides are more susceptible to unfolding under thermal conditions, which may consequently affect their biological activity.

### 2.4. Molecular Insights into Decavanadate–Lipopeptide Interactions

**Figure 5:**
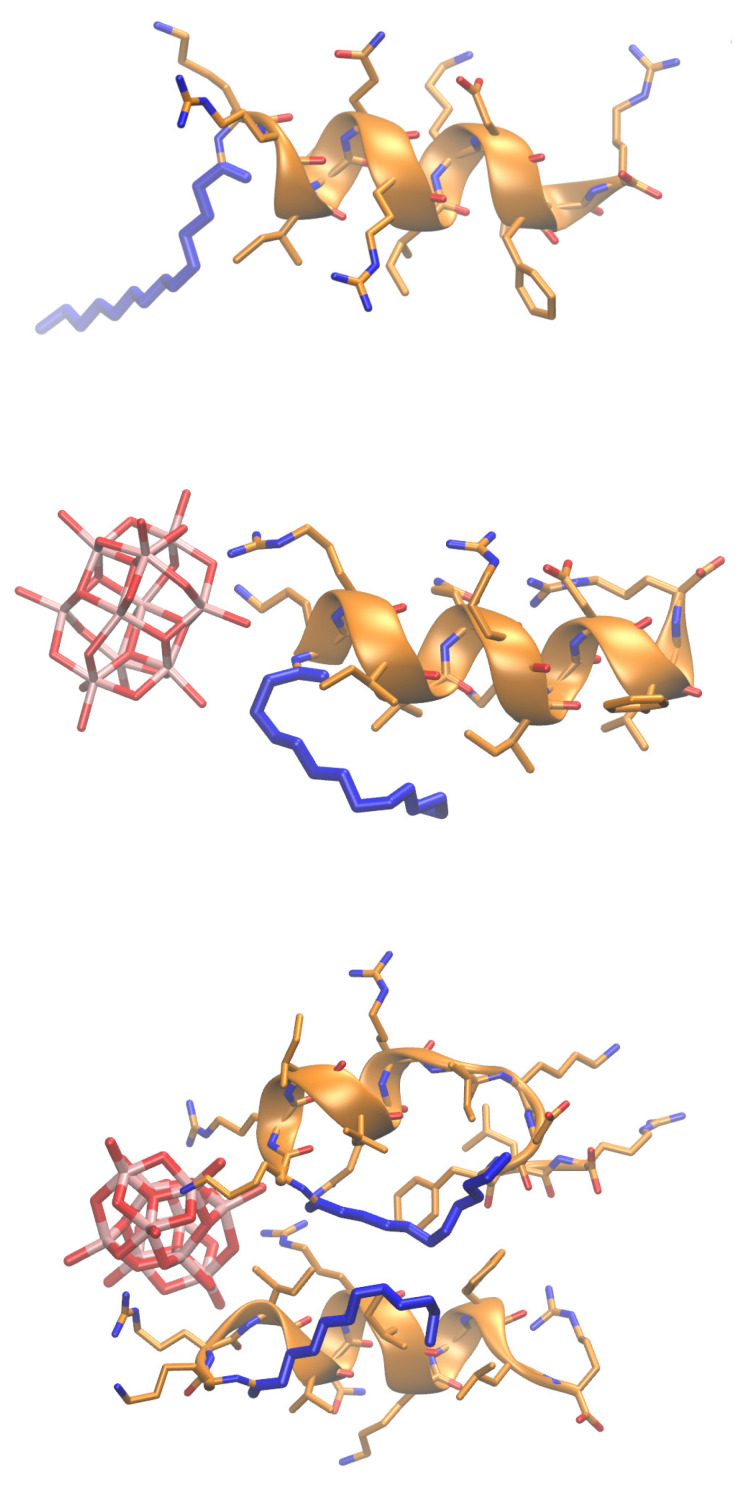
MD-based analysis of C14-KR12’s interactions with decavanadate. Top panel: unbound C14-KR12; middle and bottom panels: representative structures of one and two C14-KR12 peptides binding a decavanadate ion. The myristate chain conjugate is shown as thick blue sticks, positively charged residues of KR12 and a decavanadate ion are shown as sticks, and the secondary structure of the KR12 peptide is rendered as a cartoon.

MD-based analysis was performed to enhance understanding and verify the experimental results concerning the intermolecular interaction modes of lipopeptides with decavanadate. The results obtained for the C12-KR12 and C14-KR12 peptides were very similar. Therefore, only the data for the C14-KR12 peptide are presented. The results are fully consistent with the experimental ITC data, indicating that the binding of decavanadate ions to the peptide is predominantly governed by electrostatic interactions with the side chains of arginine and lysine residues (Figure 5 and Supplementary Figure S1). Furthermore, it also supports previously published findings identifying electrostatic interactions as the primary driving force for the binding of different types of POMs to proteins, both in solution and in the solid state [22].

It is noteworthy that among several arginine and lysine residues, Lys18 and Arg19 were found to be the most probable contributors to the binding for both 1:1 and 2:1 peptide–decavanadate stoichiometry. In the system with 1:2 stoichiometry, only one decavanadate ion was bound, while another was repulsed due to its highly negative charge. Interestingly, the aliphatic tail of the peptide did not participate in binding. Instead, it folded to the hydrophobic patch of the peptide for both lipopeptides in all simulated molecular complexes. These intramolecular interactions could contribute to the stabilization of the α-helical structure of the peptide that was maintained through the MD simulation. It would be expected that a longer tail would contribute more to this kind of stabilization of the helix because of its bigger size. This finding is consistent with the conclusions drawn from CD spectroscopic analysis regarding the influence of hydrophobic chain length on the α-helical structure content of the lipopeptides.

**Figure 6:**
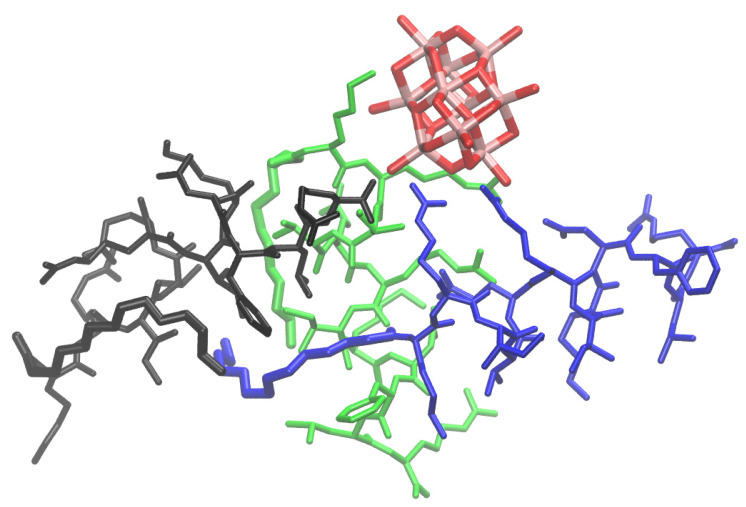
MD-based analysis of C14-KR12’s interactions with decavanadate corresponding to the 3:1 stoichiometry. The molecules are rendered in sticks: each peptide derivative is in a different color, and the decavanadate ion in the atom is in a specific color.

Furthermore, we modeled the system corresponding to the experimentally most probable 3:1 stoichiometry of the peptide derivative–decavanadate complex. The obtained complex (Figure 6) suggests rather unspecific binding of the decavanadate driven by the electrostatic interactions maintained with the positively charged residues of the peptide. For two peptides (in blue and green in Figure 6) that interact via their N-terminal parts, the free energy contributions obtained by the linear interaction energy approach are significantly more favorable than for the third peptide (in black in Figure 6). These results are consistent with the data obtained for the systems with stoichiometries of 2:1 and 1:1 (see Figure 5). During the MD simulations, a variety of complex conformations was observed, with the participation of the two peptides’ N-terminals having a predominant impact on the establishment of the complex. Therefore, our model further supports and is indirectly verified by the ITC experimental data.

To further investigate the potential effect of the negatively charged Caco^−^ ion (a Brönsted base and a component of the cacodylate buffer solution) on the interaction of the peptide with decavanadate, additional molecular dynamics (MD) simulations were performed. These included the peptide in the presence of Caco^−^ ions. In this way, it was possible to assess whether these buffer components could compete with decavanadate for positively charged side chains of arginine and lysine residues and indirectly influence the interactions studied. The data obtained showed that the binding interactions between the peptide and Caco^−^ are negligible compared to those between the peptide and decavanadate. This is attributed to a substantial 6-fold difference in the net charge between the competitors, namely Caco^−^ and [V_10_O_28_]^6−^ (Supplementary Figure S1).

## 3. Materials and Methods

### 3.1. Reagents

Ammonium metavanadate (CAS: 7803-55-6, ACS reagent, ≥99.0%), and sodium cacodylate trihydrate (Caco, ≥98%) were obtained from Merck (Warszawa, Poland) and employed as received without further purification. Double-distilled water with conductivity not exceeding 0.18 μS cm^−1^ was used for preparations of buffer solutions.

### 3.2. Synthesis of (NH_4_)_6_[V_10_O_28_](H_2_O)_6_

The compound (NH_4_)_6_[V_10_O_28_](H_2_O)_6_ was synthesized using a general method for the preparation of ammonium decavanadate salts. A mixture of ammonium metavanadate (NH_4_VO_3_, 1.17 g) and water (30 mL) was stirred continuously at room temperature for approximately one hour. The solution was then filtered to remove any undissolved solids. To the resulting yellow solution, 50% aqueous acetic acid was gradually added to adjust the pH to 4.5. Once the appropriate pH was reached, 95% ethanol was added to the mixture while stirring on a magnetic stirrer for 30 min. The mixture was then left overnight in a refrigerator to crystallize. An orange precipitate of (NH_4_)_6_[V_10_O_28_](H_2_O)_6_ fell out, which was washed with cold 95% ethanol and then air dried at room temperature. The composition of the compound was established based on the elemental analysis of hydrogen and nitrogen (Vario EL analyzer Cube CHNS): Anal. Calcd for (NH_4_)_6_[V_10_O_28_](H_2_O)_6_: H, 3.1%, N 7.2%. Found: H, 2.9%, N 7.1%.

### 3.3. Synthesis of the C12-KR12 and C14-KR12 Peptides

The peptides C12-KR12 (dodecanoic acid-KRIVQRIKDFLR-NH_2_) and C14-KR12 (tetradecanoic acid-KRIVQRIKDFLR-NH_2_) were synthesized manually by the solid-phase method using 9-fluorenylmethoxycarbonyl (Fmoc) chemistry on resin modified by a Rink amide linker resin with loading of 1.0 mmol/g (Orpegen Peptide Chemicals GmbH, Heidelberg, Germany) according to the procedure described in our previous work [17]. Both peptides were removed from the resin, along with side chain deprotection, in a one-step procedure using a mixture of trifluoroacetic acid (TFA; Apollo Scientific, Denton, UK), triisopropylsilane (TIS; Sigma-Aldrich, St. Louise, MO, USA), and water (95:2.5:2.5 *v*/*v*/*v*) [17]. Finally, the peptides were purified by solid-phase extraction (SPE) on Isolute TM SPE columns (flash, C18, 25 mL) [23,24].

The purity of the peptides after purification was at least 95%, as determined by analytical reversed-phase high-performance liquid chromatography (RP-HPLC). Their identity was confirmed by electrospray ionization mass spectrometry (ESI-MS).

### 3.4. Isothermal Titration Calorimetry (ITC)

ITC experiments were performed at 298.15 K using an AutoITC isothermal titration calorimeter (MicroCal Inc. GE Healthcare, Northampton, UK). Reagents were dissolved directly in 50 mM sodium cacodylate buffer at pH 5. The experiments consisted of injecting 10.02 μL (29 injections, 2 μL for the first injection only) of 1 mM buffered solution of (NH_4_)_6_[V_10_O_28_](H_2_O)_6_ into the reaction cell, which initially contained the 0.25 mM buffered solution of the peptide. A background titration, consisting of an identical titrant solution but with only the buffer solution in the reaction cell, was subtracted from each experimental titration due to the heat of dilution. The titrant was injected at 240 s intervals. Each injection lasted 20 s.

### 3.5. Circular Dichroism Spectroscopy (CD)

Circular dichroism (CD) spectra were recorded in 50 mM sodium cacodylate buffer at pH 5 on a Jasco-715 automatic recording spectropolarimeter (Jasco Inc., Easton, MD, USA) over the temperature range 298.15–358.15 K. The peptide concentration was 0.1 mM, and the mixture of peptide and (NH_4_)_6_[V_10_O_28_](H_2_O)_6_ was prepared in a molar ratio of 1:1.6. The spectra were recorded in the 190–260 nm wavelength range in 1 mm quartz cuvettes (sample volume: 0.3 mL), using a sensitivity of five millidegrees and a scan speed of 50 nm min^−1^. Each spectrum was scanned three times. The percentage of a-helical structure was calculated from the mean molar ellipticity [θ] at 222 nm. Details of the CD data analysis were previously described in [25].

### 3.6. Molecular Dynamics Simulations

MD simulations were performed using the AMBER16 software package [26]. To build C12-KR12 and C14-KR12 structures, the experimental structure of the LL-37 peptide corresponding to the 18–29 residue numbers was obtained from the PDB (PDB ID: 2K6O, the 1st NMR model), and aliphatic chains were added to the N-terminus of the peptide in the Xleap module of AMBER. The partial charges and the parameters for the added aliphatic chains were calculated with the standard and AMBER-compatible RESP procedures [27] using GAUSSIAN16 [28] and obtained from the gaff parameter set [29], respectively. Charges for decavanadate [V_10_O_28_]^6–^ and Caco [(CH_3_)_2_AsO(O)^−^] were also obtained with RESP procedures compatible with the AMBER force field family, while the bond, angle, and dihedral angle parameters were obtained directly from the corresponding Gaussian 16 geometry optimization and implemented into AMBER libraries with the strong harmonic constants of 300 kcal/mol/Å and 100 kcal/mol/° for bond and angle, respectively, due to the absence of these force constants in the gaff parameter set. Eight types of molecular systems were simulated: C12-KR12 and C14-KR12 with one and two decavanadate molecules, with one Caco molecule, and two modified peptides of each kind with one decavanadate molecule. For this, the molecules were placed randomly in Xleap. A truncated octahedron TIP3P periodic box of a 15 Å water layer from the box’s border to solute was used to solvate complexes. The charge was neutralized with Na^+^ or Cl^−^ counterions. Gaff and ff14SB [30] force field parameters were used for non-peptidic and peptidic parts of the molecular system, respectively. Energy minimization was carried out in two steps: beginning with 500 steepest descent cycles and 10^3^ conjugate gradient cycles with 100 kcal/mol/Å^2^ harmonic force restraints, and continuing with 3 × 10^3^ steepest descent cycles and 3 × 10^3^ conjugate gradient cycles without any restraints. Following minimization steps, the system was heated up from 0 to 300 K for 10 ps with harmonic force restraints of 100 kcal/mol/Å^2^. Subsequently, the system was equilibrated at 300 K and 10^5^ Pa in the isothermal isobaric ensemble for 500 ps. Both temperature and pressure were equilibrated in the system after these steps. Afterward, the productive MD run was carried out in the same isothermal isobaric ensemble for 100 ns, which was sufficient to extensively sample the conformational space of the simulated complexes. The particle mesh Ewald method for treating electrostatics and the SHAKE algorithm for all the covalent bonds containing hydrogen atoms were implemented in the MD simulations. The equilibration criteria were the stability of the obtained complex structure in terms of the RMSD (root mean square deviation), which was already achieved after a couple of nanoseconds.

## 4. Conclusions

Two complementary experimental methods, isothermal titration calorimetry (ITC) and circular dichroism (CD) spectroscopy, supported by in silico analysis, have been used to rigorously characterize the interactions between lipopeptides and decavanadate ions ([V_10_O_28_]^6−^). The selected cationic lipopeptides, which are conjugates of the KR12 peptide with lauric acid (C12-KR12) and myristic acid (C14-KR12), serve as excellent models for the study of non-covalent interactions with decavanadates due to their relatively small size compared to proteins. The results indicate that the affinity of the cationic lipopeptides for decavanadates is mainly driven by enthalpic factors resulting from electrostatic interactions and hydrogen bonding. Molecular dynamics (MD) simulations confirmed the experimental results, showing that the binding of decavanadate ions to the peptide occurs primarily via electrostatic interactions with the side chains of the Lys^18^ and Arg^19^ residues. Under the experimental conditions (50 mM cacodylate buffer, pH 5), the peptides adopt an α-helical conformation, with the peptide containing the longer hydrocarbon chain fragment (the myristoyl group, C14-KR12) showing greater thermal stability. As a result of lipopeptide–decavanadate interactions, the α-helical structure of cationic peptides is disrupted. These interactions also lead to a reduction in the thermal stability of their secondary structure. Consequently, this phenomenon can affect the biological functions of biomolecules containing positively charged amino acid residues.

## Figures and Tables

**Table 1:** Thermodynamic parameters of binding interactions of C12-KR12 and C14-KR12 with [V_10_O_28_]^6−^ in the 50 mM sodium cacodylate buffer with a pH of 5, at 298.15 K (standard deviation values in parentheses).

Parameter	[V_10_O_28_^6−^] + C12-KR12	[V_10_O_28_^6−^] + C14-KR12
*N* (stoichiometry)	0.37 (±0.01)	0.39 (±0.01)
log*K*_(ITC)_	6.84 (±0.15)	6.16 (±0.15)
Δ*G*_(ITC)_ [kcal mol^−1^]	−9.33 (±0.20)	−8.41 (±0.18)
Δ*H*_(ITC)_ [kcal mol^−1^]	−10.58 (±0.15)	−11.54 (±0.25)
*T*Δ*S*_(ITC)_ [kcal mol^−1^]	−1.25	−3.13

**Table 2:** Temperature-dependent α-helical content of C12-KR12 and C14-KR12 in 50 mM sodium cacodylate buffer (pH 5) with and without [V_10_O_28_]^6−^.

Temperature [K]	The Percentage of α-Helical Content [%]			
	C12-KR12	[V_10_O_28_]^6−^:C12-KR12	C14-KR12	[V_10_O_28_]^6−^:C14-KR12
298.15	36	19	49	21
308.15	32	18	46	19
318.15	26	7	42	19
328.15	7	5	38	8

## Data Availability

The data presented in this study are available on request from the corresponding author.

## Supplementary Materials

The following supporting information can be downloaded at: https://www.mdpi.com/article/10.3390/molecules30071589/s1, Figure S1: Distances between decavanadate (left panel) and Caco- ions (right panel) to the side chain charged groups of C14-KR12 in the corresponding MD simulations. Top panels: distances in the course of the MD simulation; bottom panel: density of the probability for the distances obtained from the whole trajectory.
