# Supplementary material for: Interactions of Laurylated and Myristoylated KR12 Fragment of the LL37 Peptide with Polyoxidovanadates
Source: Molecules. 2025 Apr 2;30(7):1589. doi: 10.3390/molecules30071589 (PMC11990403; doi:10.3390/molecules30071589)
Supplement: Supplementary file 1 [file molecules-30-01589-s001.zip › molecules-3510994-supplementary.pdf]

molecules

MDPI

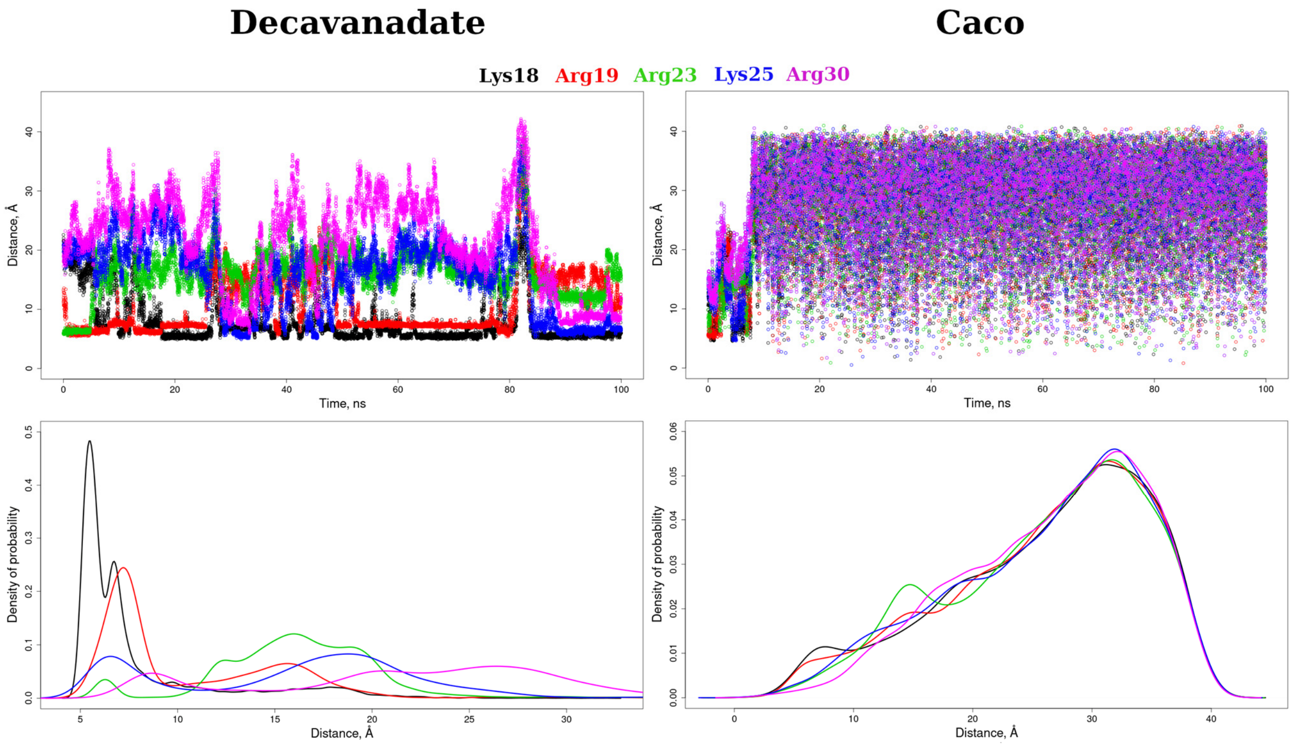

Figure S1. Distances between decavanadate (left panel) and Caco$^-$ ions (right panel) to the side chain charged groups of C14-KR12 in the corresponding MD simulations. Top panels: distances in the course of the MD simulation; bottom panel: density of the probability for the distances obtained from the whole trajectory.
